# Influence of Psychological Stress on the Response to Periodontal Treatment: Protocol for a Systematic Review

**DOI:** 10.2196/56765

**Published:** 2024-11-12

**Authors:** Kelly R Vargas Villafuerte, Luiz H Palucci Vieira, Karina Oliveira Santos

**Affiliations:** 1 Grupo de Investigación en Salud Integral Humana (GISIH) Facultad de Ciencias de la Salud Universidad César Vallejo (UCV) Lima Peru; 2 Department of Biological Sciences Bauru School of Dentistry University of São Paulo (USP) Bauru, São Paulo Brazil

**Keywords:** periodontal disease, periodontal therapy, integral health, oral health, evidence-based, periodontitis, inflammatory diseases, inflammatory response, anxiety, stress, depression, periodontal health

## Abstract

**Background:**

The interaction between stress and periodontal treatment is a topic of growing interest. It stands out as a field of research that sheds light on the complexity of oral health in individuals subjected to high levels of emotional stress.

**Objective:**

This paper aims to provide a protocol for a systematic review to examine the scientific evidence related to the influence of psychological stress on the response to periodontal treatment.

**Methods:**

The Preferred Reporting Items for Systematic Reviews and Meta-Analyses (PRISMA) guidelines will be followed, and the study is based on a formulation of PECO (Participants, Exposure, Comparators, and Outcomes) questions. This systematic review will involve a literature search covering studies published from January 2000 to May 2024. It will include searching the PubMed, Web of Science, EBSCOHost, Scopus, and ProQuest databases to identify relevant studies. After selection, data extraction and quality assessment (using the Ottawa-Newcastle Scale) of the included studies will be carried out. The objective is to integrate high-quality evidence on how psychological stress impacts the outcomes of periodontal treatment. Depending on the number and methodological consistency of included studies, the results may be presented by meta-analysis or qualitative synthesis.

**Results:**

The current stage of the study consists of selecting studies for the systematic review as outlined by this protocol. The search, screening, and data extraction began in January 2024. The final results are expected by July 2024, with final manuscript submission planned for November 2024.

**Conclusions:**

This review will help clarify how psychological factors, such as stress, influence the results of periodontal treatment, providing valuable guidelines for future research and helping clinicians make decisions about the best treatment options for patients.

**Trial Registration:**

Open Science Framework (OSF) Registries qfs6p; https://osf.io/qfs6p

**International Registered Report Identifier (IRRID):**

PRR1-10.2196/56765

## Introduction

Psychological stress represents an emotional and physiological response of the body to adverse stimuli, which occurs when a person perceives a situation or event as threatening to his or her coping resources [1]. It can be derived from various sources in everyday life, such as work problems, personal relationships, and adverse economic situations, and these stressors influence well-being, behavior, and general health [2]. In its acute manifestation, its effect originates in the immune system [2,3], while in its chronic form, it can lead to prolonged inflammatory processes that negatively impact both the immune and endocrine systems [4]. These effects contribute to the development of diseases such as diabetes mellitus, cardiovascular diseases, cold sores, lichen planus, and periodontitis [4-6].

Periodontitis is a chronic inflammatory disease that affects the supporting tissues of the teeth. In its development and progression, dysbiosis (imbalance in the microbiota) and the host’s immune-inflammatory response play fundamental roles [7]. Research has indicated a positive relationship between psychological stress and periodontal diseases [2,8-10]. Stress can trigger changes in the immune system [2], characterized by an increase in the inflammatory response and a decrease in the body’s immune defenses [3]. The presumed biological mechanisms affect the activity of stress axes, namely, the hypothalamic-pituitary-adrenal axis and the sympathetic nervous system, leading to a subsequent dysregulation of the immune system [6]. In addition, it has been observed that stress can lead to a reduction in saliva flow and its acidification, which, in turn, may increase the prevalence of periodontal diseases [8,9].

Simultaneously, research has also demonstrated that stress has an adverse effect on periodontal treatment [2,11,12] and delays healing [13], indicating that stressed individuals do not show a reduction in periodontal clinical parameters [11]. Studies also suggest that stress triggers the production of substances such as cortisol, dehydroepiandrosterone [14], and catecholamines [3,4], which can modulate bacterial growth in periodontal disease and the expression of virulence factors [4]. In addition, factors like stress and anxiety can induce behavioral changes, such as poor oral hygiene, low-quality sleep, increased smoking [2,3], as well as an inadequate diet [9] that includes foods rich in carbohydrates, sugar, and acids, further exacerbating periodontitis [9]. In this context, it seems crucial to consider and address psychological factors as an integral part of periodontal therapy [15].

Currently, there are systematic reviews on the impact of psychological factors, such as stress and anxiety, on periodontal health [16,17], as well as on stress and depression as risk factors for periodontal diseases [8,18]. However, systematic reviews addressing how psychological stress specifically affects the outcomes of periodontal treatment have not yet been conducted. Therefore, it is imperative to delve into this aspect and understand how this factor may influence periodontal treatment; that is, the impact of psychological factors such as stress on periodontal treatment constitutes a highly relevant and complex aspect that demands a comprehensive approach to understanding how stress affects the immune response and patient behavior, which may influence the severity of periodontitis [5] and in the recovery capacity of periodontal tissue [13,14].

The objective of this paper is to provide a protocol for a systematic review intended to evaluate the influence of stress on the clinical outcome of periodontal treatment. The addressed PECO (Participants, Exposure, Comparators, and Outcomes) question will focus on “What is the influence of psychological stress on the response to periodontal treatment?”

## Methods

### Registration

This protocol was preregistered in the Open Science Framework (OSF) Registries (ID: qfs6p) [19]. This protocol, as presented in its entirety below, follows the 2015 PRISMA-P (Preferred Reporting Items for Systematic Review and Meta-Analysis for Protocols) checklist (Multimedia Appendix 1) [20]. It is also intended that this systematic review will contain the items of the PRISMA (Preferred Reporting Items for Systematic Reviews and Meta-Analyses) guidelines, 2020 version [21].

### Electronic Databases and Search Strategy

The search will include all articles indexed in the PubMed, Web of Science Core Collection (Clarivate), EBSCOHost, Scopus, and ProQuest databases published in the English language from January 2000 to May 2024. In addition, we will also consider an additional exploration of the reference lists and bibliographies of all potential full-text articles. EndNote software (version X7.0.1; Clarivate) will be used to facilitate reference management.

In Table 1, the search terms are presented exactly as they will be used in each specific database. A Boolean search strategy was elaborated, considering also those key terms previously used in existing systematic reviews [8,16]. For the PubMed database, Medical Subject Headings terms were used to ensure a more comprehensive and precise search.

**Table 1:** The search strategy used exactly as entered into each specific database considered in this review. The PubMed strategy included MeSH (Medical Subject Headings) terms.

Database	Search strategy
PubMed	(((((((periodont*[Text Word]) OR periodontal therapy[MeSH Terms]) OR non-surgical periodontal therapy[MeSH Terms]) OR scaling[Text Word]) OR periodontal debridement[Text Word]) OR mechanical debridement[Text Word]) OR plaque removal[Text Word]) AND (((((stress[Text Word]) OR psychological stress[MeSH Terms]) OR emotional stress[Text Word]) OR chronic stress[Text Word]) OR job-related stress[Text Word]) AND (((control*[Text Word]) OR baseline[Text Word]) OR *stressed group*[Text Word]) AND (((probing depth[Text Word]) OR clinical attachment level[Text Word]) OR bleeding of probing[Text Word])
Web of Science	Periodont* OR Periodontal therapy OR Non-surgical periodontal therapy OR Scaling OR Periodontal debridement OR Mechanical debridement OR Plaque removal (Topic) AND Stress OR Psychological stress OR Emotional stress OR Chronic stress OR Job-related stress (Topic) AND Control* OR Baseline OR *Stressed group* AND Probing depth OR Clinical attachment level OR Bleeding of probing (Topic)
EBSCOHost	(Periodont* OR Periodontal therapy OR Non-surgical periodontal therapy OR Scaling OR Periodontal debridement OR Mechanical debridement OR Plaque removal) AND (Stress OR Psychological stress OR Emotional stress OR Chronic stress OR Job-related stress) AND (Control* OR Baseline OR *Stressed group*) AND (Probing depth OR Clinical Attachment level OR Bleeding of probing)
Scopus	(TITLE-ABS-KEY (“periodont*” OR ”periodontal therapy“ OR ”non-surgical periodontal therapy“ OR scaling OR ”periodontal debridement“ OR ”mechanical debridement“ OR ”plaque removal“) AND TITLE-ABS-KEY (stress OR ”psychological stress“ OR ”emotional stress“ OR ”chronic stress“ OR ”job-related stress“) AND TITLE-ABS-KEY (control* OR baseline OR ”*stressed group*“) AND TITLE-ABS-KEY (”probing depth“ OR ”clinical attachment level“ OR ”bleeding of probing“))
ProQuest	noft(Periodont* OR Periodontal therapy OR Non-surgical periodontal therapy OR Scaling OR Periodontal debridement OR Mechanical debridement OR Plaque removal) AND noft(Stress OR Psychological stress OR Emotional stress OR Chronic stress OR Job-related stress) AND noft(Control* OR Baseline OR Stressed group*) AND noft(Probing depth OR Clinical Attachment level OR Bleeding of probing)

### Inclusion and Exclusion Criteria

#### Type of Studies to be Included

Cohort or cross-sectional studies, case-control studies, and studies published in English are considered eligible. A targeted research query designed to enhance the literature search was formulated, outlining the assigned PECO measures as shown in Textbox 1.

**PECO (Participants, Exposure, Comparators, and Outcomes) measures.**

- Participants (P): Adult patients diagnosed with periodontitis and undergoing periodontal treatment (prophylaxis and scaling and root planing [ultrasonic devices, curettes, and polishing]).
- Exposure (E): Psychological stress (assessed by psychometric instruments, validated questionnaires to measure stress levels, or studies that used biomarkers to assess stress [eg, salivary cortisol]).
- Comparators (C): Patients with periodontitis without psychological stress (assessed by psychometric instruments, validated questionnaires to measure stress levels, or studies that used biomarkers to assess stress [eg, salivary cortisol]).
- Outcomes (O): Clinical outcome of periodontal treatment.
  Primary outcomes:
- Changes in probing pocket depth (PPD) and clinical attachment level (CAL) were measured in millimeters.
- Changes in bleeding on probing (BOP) are measured in percentage.
  Secondary outcomes
- Changes in plaque index (PI) are measured in percentage.

Variables that will be considered for extraction include, for example, probing pocket depth, clinical attachment level, bleeding on probing, and plaque index. Null-hypothesis significance tests may include independent-samples *t* test, Mann-Whitney *U* test, Kruskal-Wallis test, analysis of covariance (ANOVA) with post hoc, linear regression models, logistic regression models, and multivariable ANCOVA. For this review synthesis, the threshold for the significance will be preset at *P*≤.05.

#### Type of Studies to be Excluded

The following types of studies will be excluded: observational studies (without mechanical debridement or professional plaque removal); studies where patients received other adjuvant therapies (eg, laser, probiotics, or antibiotic use) or any type of periodontal treatment in the last 6 months; studies on patients with systemic or autoimmune disorders, which influence treatment outcomes such as diabetes; studies on patients who are pregnant or breastfeeding; studies on patients using immunosuppressive drugs or drugs that affect the oral microbiome (eg, antineoplastic or antiepileptic drugs); studies that did not go through ethics committee; letters, case reports, and short communications; and studies in animal models and in vitro studies.

### Selection of Studies

The selection process will be in accordance with PRISMA guidelines. Data collection will be carried out using a specific spreadsheet, with parameters selected after a pilot study involving approximately 10 included studies. In addition, 2 reviewers, identified as participating researchers, will conduct the assessment independently. In case of disagreement, a third researcher with expertise in the field will resolve discrepancies.

If there is unclear or omitted information from the selected studies, such as missing data or incomplete text, the authors will be contacted by email, and weekly attempts will be made for a maximum of 5 weeks. If there is no response from the authors, the study will be excluded.

Studies meeting the inclusion criteria will undergo validation and data extraction.

### Data Extraction and Evidence Synthesis

The relevant data extracted from each study will describe the designated PECO measures: author’s name and date of publication; country; study design; participant characteristics; periodontal treatment; definition of psychological stress; stress diagnosis (stress scale or biomarkers used); clinical parameters (probing depth, clinical attachment level, bleeding on probing, and plaque index); monitoring and outcome measures of interest to the review (regular clinical monitoring to monitor the effectiveness of treatment); authors’ conclusions; and source of funding.

Subsequently, the “best evidence synthesis method” will be applied to classify the level of evidence [22]. It will be considered strong (consistent findings observed among multiple high-quality studies), moderate (consistent findings observed among multiple moderate-quality studies and/or 1 high-quality study), limited (findings provided by 1 moderate-quality study and/or only low-quality studies), conflicting (when inconsistent findings were observed), or “no evidence”(when there were no available studies) [22]. Consistency will be defined as ≥75% of studies report results in the same direction, and inconsistency will be defined as <75% of studies report results in the same direction.

### Methodological Quality and Risk of Bias Assessments

A total of 2 authors (KRVV and KOS) will independently evaluate the methodological quality of each included study using the Newcastle-Ottawa Scale [23]. This scale is specifically designed for nonrandomized studies. The authors will use a “star” system for each study, considering 3 main components: (1) quality of study participant selection, (2) comparability, and (3) exposure and outcome. The maximum score is 9 points for case-control studies and 10 points for cross-sectional studies. The total score will categorize the studies into three groups: (1) high quality (total score: 7 to 9 or 10), (2) moderate quality (total score: 4 to 6), and (3) low quality (total score: 0 to 3). To determine consistency, the interrater agreement will be calculated using the Cohen κ coefficient.

Furthermore, the risk of bias in the outcomes or interpretations will be independently determined for each included study. This will be done using the Risk of Bias Assessment Tool for Non-Randomized Studies [24]. Each element will be evaluated as presenting low, high, or unclear risk concerning participant selection criteria, potential confounding variables, accuracy of exposure measurement, blinding of outcome assessments, handling of incomplete outcome data, and selective outcome reporting.

### Data Synthesis

If there are a sufficient number of studies and no substantial variations in the methods used among them, the results may also be presented through a meta-analysis (quantitative synthesis). Regardless of whether the final manuscript meets the criteria for a quantitative synthesis, a qualitative synthesis will be conducted using the best evidence synthesis method. In addition, a table with the main characteristics of the studies will be included.

We will also consider the statistical analyses used in the selected studies, including different types of regression models to adjust for confounding variables. This may include multiple linear regression for continuous dependent variables, multiple logistic regression for binary stages, and multivariable ANCOVA. In addition, we will also include other statistical analyses, such as clarification analysis, as applicable.

## Results

The search, screening, and data extraction began in January 2024, following the established protocol. From the initial search to find previous reviews on the influence of psychological stress on periodontal treatment, no reviews were identified that had this objective, although we did find reviews on the association of psychological stress on periodontitis and about stress as a risk factor for periodontitis (Tables 2 and 3).

The results are expected to be completed by July 2024, and the final manuscript of the systematic review is anticipated to be submitted in November 2024.

**Table 2:** Some review studies that potentially address the influence of stress on periodontal treatment.

Reference	Type of review	Guidelines	Date of searches	Databases considered
Peruzzo et al [18]	Systematic	—^a^	January 1, 1990, to April 1, 2006	MEDLINE and the Cochrane Oral Health Register
Botelho et al [25]	Systematic and meta-analysis	PRISMA^b^	Up to September 2017	Electronic general, open access, regional, and gray literature databases
Decker et al [8]	Systematic	PECOS^c^ framework	Up to December 2017	MEDLINE (OVID), Embase (OVID), and CENTRAL (Cochrane Library); gray literature at the New York Academy of Medicine Grey Literature Report
Castro et al [16]	Systematic	PRISMA	Until March 2018	PubMed, Scopus, Web of Science, Lilacs, and Cochrane Library; Google Scholar and OpenGrey were used as gray literature sources
Badia et al [26]	Systematic and meta-analysis	PRISMA	Until June 2021	MEDLINE, Embase, and the Cochrane Library
Aggarwal et al [17]	Systematic and meta-analysis	—	Until December 2019	PubMed, Embase, and Scopus

^a^Not available.

^b^PRISMA: Preferred Reporting Items for Systematic Reviews and Meta-Analyses.

^c^PECOS: Participants, Exposure, Comparators, Outcomes, and Study Design.

**Table 3:** Results of the initial search for review studies that potentially address the influence of stress on periodontal treatment.

Reference	Year	Aim	Results
Peruzzo et al [18]	2007	Review the evidence on the influence of stress and psychological factors on periodontal disease	In total, 8 (57.1%) studies found a positive outcome between psychosocial factors of stress and periodontal disease, 4 (28.5%) studies observed a positive outcome for some characteristics and a negative outcome for others, and 2 (14.2%) studies found a negative outcome between psychosocial factors of stress and periodontal disease.
Botelho et al [25]	2018	To systematically assess whether periodontitis has a meaningful effect on salivary cortisol, reflecting changes in free blood cortisol levels	A network meta-analysis was performed comparing salivary cortisol response between chronic and aggressive periodontitis patients. Although the indirect estimates were not statistically significant, the results were consistent with the Bucher test (*P*=.99) and favored aggressive periodontitis regarding salivary cortisol response. Overall, the salivary cortisol response in patients with aggressive periodontitis is, on average, 42% higher than in patients with chronic periodontitis (mean ratio 1.42 95% CI 0.97-2.06; *P*=.99).
Decker et al [8]	2020	To evaluate the impact of stress-related disorders on the progression of periodontal disease and evaluate the growing body of evidence of stress as a risk indicator for periodontal disease progression	Relationships between stress-related disorders and serum and salivary biomarkers such as cortisol, dehydroepiandrosterone, chromogranin A, and proinflammatory cytokines were identified.
Castro et al [16]	2020	To explore the relationship between psychological stress and periodontitis by analyzing cortisol levels and periodontal clinical parameters	3 articles were selected by full text. Among them, 2 articles reported a positive association between psychological stress and periodontitis. All articles were classified as low risk of bias. Even though 2 articles highlighted an association between psychological stress and the presence of a possible modulatory pattern of cortisol levels in clinical parameters of periodontitis, more studies are necessary to elucidate this question.
Badia et al [26]	2022	To evaluate the literature on the impact of psychological stress on periodontitis and its progression and to evaluate current evidence of psychological stress as a risk factor for periodontal disease using psychological questionnaires	The results of the meta-analysis did not show statistically significant differences between the 2 groups. There is a relationship between psychological stress and the severity of periodontitis. This relationship must be considered a possible risk factor.
Aggarwal et al [17]	2022	To assess the association between psychologic stress, anxiety, and periodontitis	A total of 25 studies were selected for systematic review, and only 14 studies could be used for meta-analysis in 3 subsets. The pooled odds ratio for stress and periodontitis was 1.78, which was statistically highly significant (*I*^2^=98.6%, *P*=.00). Mean salivary cortisol levels as a measure of stress in patients with periodontitis was 4.81 nmol/L (*I*^2^=98%, *P*=.08). State-Trait Anxiety Inventory score was –1.28 (*I*^2^=0%, *P*=.06) for state anxiety and –0.11 (*I*^2^=0%, *P*=.85) for trait anxiety in patients with periodontitis.

## Discussion

### Principal Findings

Studies have shown a correlation between psychological stress and various inflammatory diseases [27,28]. Research in the field of dentistry shows systematic reviews that explore the association between psychological stress and periodontitis [16,17,25] or psychological stress as a possible risk factor for periodontal diseases [8,18,26]. However, to date, no systematic review has focused on understanding how psychological stress specifically influences periodontal treatment outcomes.

There is evidence that psychological stress affects the body’s inflammatory response [29,30], which together may increase susceptibility to periodontal disease [16,17,31,32] and hinder adherence to periodontal treatment, resulting in slower healing and less favorable clinical outcomes [32,33]. Therefore, it is hypothesized that the results of the selected studies confirm a significant influence of psychological stress on periodontal treatment. Patients with high levels of stress are likely to show worse periodontal treatment outcomes compared with those with lower levels of stress. A study [34] evaluated the effects of psychological stress on periodontitis healing in rats and the contribution of baseline fibroblast growth factor expression to the healing process; it observed that psychological stress could delay periodontitis healing, which may be mediated in part by down-regulation of baseline fibroblast growth factor expression in the periodontal ligament. Therefore, it is important to investigate whether patients with elevated levels of psychological stress may require personalized, lifestyle-changing interventions to optimize treatment outcomes.

A strength of this review is the rigorous methodology, which reinforces the validity of the results. In addition, this review will provide an updated, specific, and detailed view of how psychological stress directly affects periodontal treatment outcomes, highlighting the importance of considering psychosocial factors. However, there may be limitations to consider, such as variability in the measurement of psychological stress among the included studies, which may introduce bias.

For future research, it would be useful to explore the effectiveness of psychological interventions integrated into periodontal management to improve clinical outcomes. In addition, this review will identify other knowledge gaps, encouraging further studies to fill these gaps and thus improving the overall understanding of the topic, which will benefit clinical practice in the future.

To maximize the impact of these findings, several dissemination strategies are planned. These include publication in high-impact scientific journals and collaborations with dental professional societies to promote the integration of these findings into clinical practice. In addition, educational materials and workshops for oral health professionals will be developed, highlighting the importance of addressing psychological stress in periodontal treatment.

### Conclusions

This review will provide current information on how psychological stress affects the outcome of periodontal treatment, the results of which can serve as a basis for creating broader health strategies aimed at addressing psychosocial factors in promoting oral health.

## Appendix

Multimedia Appendix 1 PRISMA-P (Preferred Reporting Items for Systematic Review and Meta-Analysis for Protocols) checklist.

## Abbreviations

ANCOVA: analysis of covariance
OSF: Open Science Framework
PECO: Participants, Exposure, Comparators, and Outcomes
PRISMA: Preferred Reporting Items for Systematic Reviews and Meta-Analyses
PRISMA-P: Preferred Reporting Items for Systematic Review and Meta-Analysis for Protocols
